# A multi-centre evaluation of malignant odontogenic tumours in Nigeria

**DOI:** 10.11604/pamj.2019.33.18.16179

**Published:** 2019-05-10

**Authors:** Olujide Oladele Soyele, Olajumoke Ajibola Effiom, Ahmed Oluwatoyin Lawal, Mark Chukwuemeka Nwoga, Kehinde Emmanuel Adebiyi, Adetayo Aborisade, Abiodun Saheed Olatunji, Adetokunbo Babajide Olawuyi, Adeola Mofoluwake Ladeji, Robinson Obos Okiti, Henry Ademola Adeola

**Affiliations:** 1 Department of Oral Maxillofacial Surgery and Oral Pathology, Obafemi Awolowo University, Ile-Ife, Nigeria; 2 Department of Oral and Maxillofacial Pathology and Biology, College of Medicine, University of Lagos, Lagos, Nigeria; 3 Department of Oral Pathology, College of Medicine, University of Ibadan, Ibadan, Nigeria; 4 Oral Pathology Unit, Department of Oral and Maxillofacial Surgery, Faculty of Dentistry University of Nigeria, Enugu, Nigeria; 5 Department of Oral Pathology and Oral Medicine, Faculty of Dentistry, Lagos State University College of Medicine, Ikeja, Lagos, Nigeria; 6 Department of Oral and Maxillofacial Surgery and Oral Pathology, Obafemi Awolowo University Teaching Hospital Complex, Ile-Ife, Nigeria; 7 Department of Oral and Maxillofacial Pathology and Biology, Lagos University Teaching Hospital, Lagos, Nigeria; 8 Department of Oral Pathology, University College Hospital, Ibadan, Nigeria; 9 Department of Oral and Maxillofacial Pathology, Faculty of Dentistry, University of the Western Cape and Tygerberg Hospital, Cape Town, South Africa; 10 Division of Dermatology, Department of Medicine, Faculty of Health Sciences and Groote Schuur Hospital, University of Cape Town, Cape Town, South Africa

**Keywords:** Odontogenic tumour, malignant, Nigeria, multi-centre, head and neck

## Abstract

**Introduction:**

odontogenic tumors originate from neoplastic transformation of the remnants of tooth forming apparatus. There are varying degrees of inductive interactions between odontogenic ectomesenchyme and epithelium during odontogenesis, leading to lesions that vary from benign to malignant. Malignant odontogenic tumours (MOTs) are very rare and are classified according to embryonic tissue of origin. Recently, there has been a few changes to the classification of MOTs according to the World Health Organization's (WHO) classification in 2017. This study aims to evaluate and reclassify MOTs, using a multi-centre approach in some major tertiary dental hospitals in Nigeria.

**Methods:**

this study reviewed the clinicopathological data on 63 cases of MOT diagnosed over 25 years in five major tertiary dental hospitals in Nigeria. All MOT cases were reclassified according to the recent revision to the 2017 WHO classification of odontogenic tumours.

**Results:**

from a total of 10,446 biopsies of oral and jaw lesions seen at the 5 study centres over the 25-year study period, 2199 (21.05%) cases were found to be odontogenic tumours (OTs), of which 63 were MOT. MOTs constituted 0.60% of the total biopsy cases and 2.86% of OTs. Odontogenic carcinomas presented with a mean age higher than odontogenic sarcomas. According to our 2017 WHO reclassification of MOTs, odontogenic carcinomas, ameloblastic carcinomas and primary intraosseous carcinomas were found to be the top three lesions, respectively. Carcinosarcomas were found to be extremely rare.

**Conclusion:**

using a multi-centre approach is a robust way to reduce diagnostic challenges associated with rare maxillofacial lesions such as MOTs.

## Introduction

Odontogenic tumours (OTs) constitute a wide range of lesions that are derivatives of tooth forming apparatus via neoplastic transformation of remnants of odontogenesis and odontogenic cyst [1]. Tooth forming apparatus and their embryogenic rest cells such as dental lamina (and its cell rests of Serres); enamel organ (reduced enamel epithelium); Hertwig epithelial root sheath (HERS) and its residue; epithelial cell rests of Malassez; dental papilla; and dental follicle, have all been described as possible sources of OTs. There is a varying degrees of inductive interaction between these embryogenic components of the developing tooth germ [2, 3]. These odontogenic remnants are capable of developing into epithelial and mesenchymal tissues. This differentiation potential forms the basis for the World Health Organization (WHO)'s classification of OTs into benign and malignant tumors [4].

Based on published literature, odontogenic tumors are rare lesions with varying frequency. While some authors have estimated its frequency of occurrence to be 1% [2, 3], others have reported higher values around 32% [3, 5]. Based on other studies, it constitutes about 4% of oral and maxillofacial biopsy specimens in oral pathology services [6, 7]. Similar to the classification of benign OTs, malignant odontogenic tumours (MOTs) are classified based on their histogenesis. They can emerge from epithelial components of odontogenesis; ectomesenchymal/mesenchmal remnants; or from mixed origin, consisting of both the epithelial and mesenchymal aspect [8]. MOTs are believed to constitute between 0-6.1% of OTs [9], hence they are extremely rare lesions that are exclusively located in the jaws. They arise within the jaws either as a primary lesion (de novo); from epithelial cystic linings; or via malignant transformation of a benign OTs, even though reports of malignant transformation of odontogenic cystic lining is rare [10]. This category of lesions present with diagnostic and therapeutic dilemma, consequent to their rarity coupled with cumbersome and complex histopathological features [9, 10]. MOTs are often locally aggressive with radical surgery being the mainstay of treatment. Histopathologically, MOTs can be carcinomas, sarcomas or carcinosarcomas; however the most common types are the carcinomas [8, 9].

The etiology of MOTs is idiopathic, even though research is ongoing to elucidate the underlying molecular pathogenetic mechanisms [10]. There is dearth of knowledge in the scientific literature regarding MOTs and most of the current information as to their origin, clinicopathological features, biological behavior, and therapeutic options are derived from case reports and a few series of published cases. This results in failure to develop standardized guidelines for management, diagnostic criteria and treatment protocols for MOTs [9]. Recently, World Health Organization in 2017 updated their classification of odontogenic tumors with addition of new entity in the carcinomatous and sarcomatous groups of MOTs, viz: sclerosing odontogenic carcinoma and odontogenic carcinosarcoma, and reclassification of metastasizing ameloblastoma from malignant epithelial carcinoma to benign neoplasm [11-14]. Despite the evolution of modern diagnostic techniques, arriving at a precise diagnosis of MOTs is still a difficult task [15]. Hence, this study represents one of the largest descriptive epidemiology of MOTs, compiled using a multi-centre approach among academic referral institutions in Nigeria. Using this approach, we determined the incidence, demographics and clinicopathological features of MOTs in sub-Saharan Africa.

## Methods

### Study design, participating centers and data sources

This was a 25 years retrospective review from five academic medical centers located in the south-western and eastern geopolitical region of Nigeria. Archived data from the Department of Oral Diagnosis and Oral Pathology academic in five major tertiary health institutions in eastern and western Nigeria were used in this study. Records of all MOT cases from University of Nigeria; Lagos State University; University of Ibadan; Obafemi Awolowo University; and University of Lagos were extracted from 1992-2017 (25 years), using a standardized data extraction format across all the five centres. All cases were reviewed independently of the previous diagnosis by two blinded oral pathologists and reclassified based on the 2017 WHO classification of odontogenic tumours. The extracted records contained demographic and clinicopathological data such as: age, site, gender distribution, duration, size, radiographic presentation and histological diagnosis, *inter alia*. Clinical and pathological characteristics of cases identified were described.

### Case selection and exclusion criteria

Cases without adequate clinical and histopathological information were excluded from the study. A total of 63 qualified cases of MOTs were selected for this study from the five participating centres. Cases were classified according to the 2017 WHO classification of odontogenic tumours. Selected cases were categorized by histological types, site of primary tumour, age and gender distribution.

### Data analysis

Data from the selected 63 cases were collated and processed using the SPSS data analysis software (*version 20.0; SPSS Inc. Chicago, IL*). Categorical variables were analyzed as frequencies and percentages, while quantitative variables were summarized as means and standard deviation.

## Results

### Case distribution

A total of 1550 (LUTH, Lagos), 261 ( UCH, Ibadan), 157 (0AUTHC, Ife), 118 (NAUTH, Enugu) and 113 (LASUTH, Lagos) odontogenic tumours were documented during the period under study from a total biopsy of 10446 cases of oral and jaw lesions which were seen from all study centres within the 25-year study period. 2199 (21.05%) cases of OTs were found, out of which 63 were cases of MOT. MOTs constituted (0.60%) of the total biopsy cases and 2.86% of OTs.

### Age distribution

The mean age of MOT in this study was 39.9 years ± 16.4, ranging from 4 and 76 years, with a peak age incidence observed in the 4^th^ decade of life; although majority of the cases were seen among the 3^rd^, 4^th^ and 5^th^ decades. Odontogenic carcinoma (OC) presented with a mean age of 40.7 ± 16.6, which is higher than that of odontogenic sarcoma (OS) and carcinosarcoma although it was not statistically significant. Mean age duration for males was 40.8 ± 13.5 and 38.7 ± 20.4 for females while the overall mean duration of the lesion prior to presentation was about 3.7 years with a range of 4 months to 16 years. The age distribution data obtained are summarised in Table 1.

**Table 1:** gender *vs* age distribution of MOTs

Decade (Years)	Male	Female	Frequency	Percentage (%)
<9	0	2	2	3.2
10-19	0	1	1	1.6
20-29	6	6	12	19.1
30-39	14	6	20	31.8
40-49	11	0	11	17.5
50-59	3	4	7	11.1
60-69	3	2	5	7.9
70-79	2	3	5	7.9
Total	39	24	63	100

Fisher's exact *p*=0.011 (significant)

### Swelling and pain

Swelling was observed consistently in all cases (100%) however about 91.1% of the lesions showed buccolingual cortical bone expansions. Pain was present in 67.7% of cases while ulceration was observed in 46%. The mean size of lesions was 11.3cm ± 5.9 at the widest diameter.

### Gender and site distribution

Malignant odontogenic tumour showed male preponderance of 39 cases (61.9%) as compared to females which were 24 cases (38.1%); with a male: female ratio of 1.6:1. We observed a marked mandibular predilection of 96.82% as compared to only 1 (1.59%) maxillary case. Left mandibular side was the most affected (n=48, 77.4%) site in our study (Table 2).

**Table 2:** site distribution of MOTs

Site	Mandible (n=62, 98.4%)			Maxilla (n=1, 1.6%)			Total
	Right Side	Left Side	Bilateral	Right Side	Left Side	Bilateral	
Frequency	10	48	4	1	0	0	63
Percentage (%)	15.9	76.2	6.3	1.6	0	0	100

### Histopathological distribution

**Figure 1:**
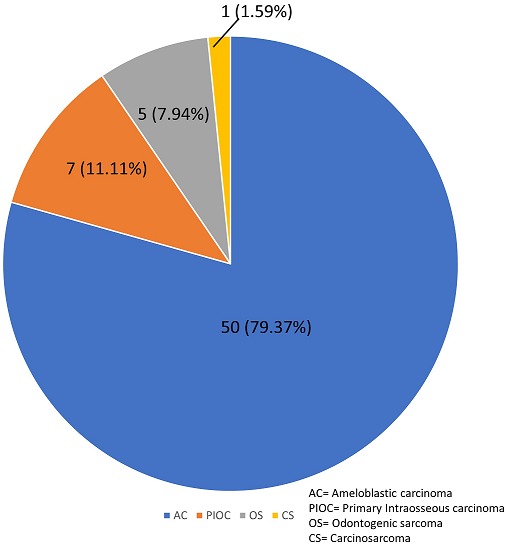
distribution of the malignant odontogenic tumours according to the 2017 World Health Organization classification

Distribution of tumor was reported according to the WHO 2017 classification (Figure 1). OC constituted 57 (90.5%) of the cases; 5 (7.9%) cases were OS; while only 1 (1.6%) case of carcinosarcoma was seen. Of the OC in this study, ameloblastic carcinoma (AC) constituted the highest frequency of 50 (87.7%) cases and 79.4% of the total MOT; while primary intraosseous carcinomas (PIOC) made up 12.3% cases of OC and 11.1% of MOT.

### Radiographic features

Generally, plain radiographs showed mixed radiolucent/radioopacity with more of multilocular appearance in areas (57.9%), while 5.3% presented a radiopaque appearance. Lytic ill-defined borders were observed in 26.2% of case.

## Discussion

Compared to the 2005 edition of the WHO classification of head and neck tumors, the current edition (published in 2017) involved important changes, which resulted from improvement and advancements in clinical and epidemiological follow-up, diagnostic immunohistochemistry, molecular biology and genetics. Hence, the current edition involved new additions, removals, and reclassification of OTs in a simpler format, eliminating subtypes that lacked clinical relevance [2, 3, 16]. Odontogenic tumors are rare lesions with varying frequency, influenced by racial distribution and geographical location; resulting in a wide range of occurrence (1-41%), according to literature [4-7]. Higher frequency of OTs was reported among Africans and Asians in a range of 3.9% to 9.6% of oral maxillofacial lesions as compared to lower frequencies (1-3%) documented in Europe and America [3-6].

MOT is a very rare subgroup of OTs. Previous studies have shown that the incidence of MOTs varies with OTs distribution (in terms of ethnics, racial and geographic variation) in the range of 0-6.1% [4, 5, 8]. In the present study, incidence of about 2.9% (n=63) of MOTs out of 2199 OTs was recorded and this is in consonance with the previous reports on rarity of this lesion. Previous studies that documented higher incidence are mostly from Africa and Asia with a range from 2.7-6.1% while those from America and Europe documented a lower range of about 1% [4 ,9, 10, 15, 17]. Contrary to the observation of a frequency of 2.9% for MOTs in our study, previous studies among Chinese population documented an incidence of 6.1% [18] and 4.7% [19]. Frequencies observed from Latin and South American studies vary between 1.2% and 2.2% [9, 20]. However in Europe, Rubini *et al.* (2017), in a 25-years retrospective study of odontogenic neoplasm among Italian population recorded an incidence of 1.1% [7]. A frequency of about 0.36% was observed in a study from the United State of America [15]. Among African studies, the incidence varies from 1.26-5.6% [10, 21-25]; justifying the global variation in the incidence of MOT. The basis for the high incidence if MOTs in people of African and Asian descent requires further research. However, MOTs have been observed to follow the global distribution of OTs.

Similar to other studies [10, 20], the overall mean age of MOTs in our study was 39.9±16.4 years (ranging from 4 to 76, and a peak age in the 4^th^ decade of life), and most cases (n=43, 68.3%) were seen within the 3^rd^, 4^th^ and 5^th^ decade of life. However, our findings were contrary (and lower) to other reports [7, 9, 26]. In the study of Rubini *et al.* (2017), 44.3±17.8 was the mean age, although with a very low case series of three MOTs; and that of Mosqueda *et al.* (2003) [9], of seven cases with 43.8 mean age and a range of 25-72 years (8); it can be inferred that the lower case series in these studies is insufficient to draw a conclusion. Also, inclusion of 'atypical ameloblastoma' with central mucoepidermoid carcinoma among MOTs could probably be responsible for the higher mean ages (of 50.29 years) obtained by Chaisuparat *et al.* (2012) [26].

Males are frequently more affected in MOTs [25], which conforms with our observation of a male to female ratio of 1.6:1 in this study. This result is similar to other reports that recorded a predominant male gender [9, 10, 21, 22]. Other studies however, observed equal gender predilection [19, 20].

Our study showed a marked mandibular involvement with a frequency of 98.4%; while the frequency for maxillary MOT lesion was 1.6%. This mandibular predominance tallies with the findings from other studies [19, 20, 26-28]; even though a maxillary predilection of 66.7% had been previously observed [7]. The reason for the high mandibular predilection may be attributed to similar observation in benign OTs such as ameloblastoma [6, 18]; and also the retention of the epithelial odontogenic rest with potential to develop into cysts or tumours in the mandible [6]. MOT can be classified into odontogenic Carcinomas (OC), odontogenic sarcoma (OS) and carcinosarcoma (CS); although OC is observed much more frequently than others [3, 4, 10, 22]. On the other hand, studies have shown that sarcomas are extremely rare malignancy accounting for about 1% of all malignancy [29]. We identified 5 (7.9%) cases of OS in our study. We recorded just one case (1.6%) of CS, showing the extent of its rarity. The reason for the low percentage of CS in our study may be due to the fact that it has not been a well recognised entity until the recent WHO 2017 classification.

OC presented with the highest value of mean age 40.7±16.6 years, compared to OS and CS which accounted for 34.6±15.9 years and 25 years, respectively. This is probably an indication that carcinomas are likely to be seen more in late adolescent/elderly. Similar difference in mean age between OC and OS was also observed in another study [20], while it is contrary to the findings of Jing *et al.* (2007) [19]. AC was the most common OC, with 50 (87.7%) cases in this study and 79.4% of all MOTs. This has equally been observed in other studies [4,8-10, 19], except for a study in Thailand where clear cell odontogenic carcinoma (CCOC) was found to be the most frequent OC and MOT, with a frequency of 70% and 46.7%, respectively. This was followed by OS which was 33.3% of MOTs [30]. CCOC and ghost cell odontogenic carcinoma (GCOC) are very rare malignant epithelial odontogenic neoplasm. CCOC was previously considered as a benign OT with biologic aggressive nature and later reclassified as a malignant lesion in WHO 2005 edition [16], based on its destructive and metastatic behaviour. GCOC is an OC with features of calcifying cystic odontogenic tumor (CCOT) and/or dentinogenic ghost cell tumor (DGCT), presenting variable histopathologic features [16, 31]. Although neither of the lesions were found in our study, Martinez *et al.* (2014) [20], however observed a CCOC frequency of 12% of the MOTs and 15.8% of OC. In addition, Jing *et al.* (2007) [19], reported the frequency of CCOC in their study as 4% of MOTs and 4.2% of OC; and GCOC as 10% and 10.4% of MOTs and OC, respectively.

AC in our study, presented with a mean age of 39.4±15.9 years, with a peak age incidence in 4^th^ decade. This result was similar to other studies [10, 19]. It is presumed that the frequency of AC would be much higher in prospective MOTs studies, and this could be due to the reclassification of malignant ameloblastoma (MA) as a benign OTs in 2017 WHO classification. Similar to AC, PIOC was also narrowed from the previous three sub-classifications in the 2005 WHO classification, to a single entity in the 2017 WHO classification [16]. It develops probably from residues of odontogenic epithelium or its rest cell, and is located within bone, without demonstrable evidence of a primary or metastatic carcinoma in other sites or from the oral or sinonasal mucosa [3]. PIOC was the second most common MOTs in our series, constituting 12.3% and 11.1% of OCs and MOTs, respectively. This is in agreement with other studies [10, 19, 20]. It presented with a mean age of 50.3±19.6 years, which concur with other studies [8, 19], but is however lower than a mean age of 56.2 years reported by Chaisuparat *et al.* (2012) [26], and 63.6±18.6 which was reported by Lawal *et al.* (2015) [10].

Sclerosing odontogenic carcinoma is a new entity that was first described by Koutlas *et al.* (2008) [32]. About 10 cases of this lesion have been reported so far [16,32]. It is a distinct entity with histopathology features of a densely sclerotic stroma, bland cytology characterized by small single-file cords and strands of epithelium, and aggressive infiltrative growth into the muscles and nerves [16]. Being a recently discovered entity, pathologists should be vigilant when encountered with a lesion of such characteristic features.

With respect to OS, it has been streamlined to a single entity based on WHO 2017 classification, as opposed to 2005 edition. Likewise, CS which was present in 1992 WHO classification, but removed in 2005 due to inadequate evidence for its existence as an entity, has been brought back to 2017 WHO classification of OTs. This was made possible by confirmatory evaluation with immunohistochemical and molecular studies on cases [3]. Previously, in 2005, odontogenic sarcomas were classified as ameloblastic fibrosarcoma and ameloblastic fibrodentinosarcoma and ameloblastic fibro-odontosarcoma, however in 2017, these malignant tumors are unified under the umbrella of odontogenic sarcomas [3, 16]. These lesions are less frequent than carcinomas as observed in this study, and supported by others [*25*, 26]. OS accounted for 7.9% of MOTs with only one case of CS reported 1.6%. Martinez *et al.* (2014), observed 24% of OS while 76% were OCs [20]. Similarly, in the study of Mosqueda *et al.* (2003), OS is composed of 14.3% and 85% were OCs [9]. In a Chinese study, the frequency of OS was found to be 4%, and OCs was 96% [19]. In addition, Dahnuthai *et al.* (2016) [30], observed a frequency of 33.3% for OS and 66.7% for OCs. However, some authors did not find any case of OS in their studies [7, 27]. The mean age of 27.3 years documented for OS in the literature [33-35], and the 34.6±14.1 reported in our study, confirm their occurrence as the most common MOT in younger patients. It was observed that CS presented with a lower mean age of 25 years, further suggesting the high possibility of occurrence of OS/CS in an adolescent than in elderly patients; although more research will be needed to corroborate this.

Although many studies on MOTs did not document the overall duration of lesion before presentation in the hospital, this study however recorded an average of 3.7 years ranging from 4 months - 16 years. This is however much higher than that recorded by Chaisuparat *et al.* (2012), of 1-2years period [26]. The wide age range is probably as a result of carcinomatous transformation of a long standing ameloblastoma. Particularly because ACs accounted for 79.4% of MOTs in this study. The disparity in the time of presentation, may also be due to the lack of education and poor infrastructure, which is common in resource limited settings [36, 37].

Swelling was consistently observed in this study with buccal-lingual cortical bone expansion in 91.1% of cases. Pain and ulceration of the oral mucosa resulting from direct tumour extension was seen in 67.7% and 46% of cases. This was similarly recorded by Chaisuparat *et al.* (2012) [26], where 16 (94.1%) cases out of 17 cases presented with buccolingual swelling; pain in 10 (58.8%) cases; and ulceration in 4 (23.5%) cases. The mean size of the lesion in this present study was 11.3±5.9 cm in its widest diameter. This is similar to 12x10 cm observed in the study of Mosqueda *et al.* (2013) [9]. These parameters (swelling, pain, ulceration) are similarly found in benign OTs, and often create diagnostic dilemma in the suspicion of malignancy. Observations from plain radiographs were either mixed radiolucent/radiopaque, and/or area of multilocular radiolucency. However, ill defined, non-corticated border was only displayed in about 26.2% of our cases. This concur with another study where about 50% of cases presented with well-defined border and multilocular radiolucency [26]. This could be a key factor in tilting consideration towards a benign lesion; as majority (73.8%) of our cases presented with a well-defined corticated margin. Hence, the need for proper clinicopathological review of these lesions in order to avoid wrong histopatological impression is essential.

Generally, treatment advocated for the proper management of these lesions is wide surgical excision followed by post-operative radiotherapy. Chemotherapy is not an effective option in treating the MOTs, although studies have suggested that the prognosis is dependent on the clinical type and location of tumour, age of the patient at diagnosis, tumour size and treatment modality [3, 35]. Advanced age at diagnosis, large tumour size, post-operative radiotherapy and OS have been found to have a negative impact on survival [3, 35].

## Conclusion

We have reviewed information on MOTs by identifying important characteristics of patients with malignant odontogenic tumors in terms of incidence and analysis of demographic with clinical presentation of these lesions from five tertiary health institutions representing the southern and eastern part of the country. The diagnosis of a malignant odontogenic tumor is complicated because of its rare presentation, limited clinical information, similarities in histopathologic observation and low index of suspicion. We believe the results of this review will throw more light and broaden the knowledge of clinicians and pathologists regarding MOTs.

### What is known about this topic

- Odontogenic tumors comprise of a heterogeneous group of maxillofacial lesions, which present with various histopathological subtypes and clinical behaviour;
- Malignant odontogenic tumours are rare maxillofacial lesions;
- Inductive interaction between odontogenic epithelium and ectomesenchyme results in the development of tumours.

### What this study adds

- This study reviews the largest cohort of malignant odontogenic tumors (MOTs) in sub-Saharan Africa (SSA) using a multi-centre approach;
- This study has employed the latest World Health Organization (WHO) classification (2017) of odontogenic tumor to reclassify MOTs in SSA;
- This study identified top ranking MOTs in Africa and made recommendations to overcome the diagnostic dilemma of delineating MOTs in resource limited settings.
